# Comparative genomics reveals the *in planta‐*secreted *Verticillium dahliae* Av2 effector protein recognized in tomato plants that carry the *V2* resistance locus

**DOI:** 10.1111/1462-2920.15288

**Published:** 2020-10-29

**Authors:** Edgar A. Chavarro‐Carrero, Jasper P. Vermeulen, David E. Torres, Toshiyuki Usami, Henk J. Schouten, Yuling Bai, Michael F. Seidl, Bart P. H. J. Thomma

**Affiliations:** ^1^ Laboratory of Phytopathology Wageningen University and Research Wageningen 6708 PB The Netherlands; ^2^ Laboratory of Plant Breeding Wageningen University and Research Wageningen 6708 PB The Netherlands; ^3^ Theoretical Biology and Bioinformatics Group, Department of Biology Utrecht University Utrecht The Netherlands; ^4^ Graduate School of Horticulture Chiba University Matsudo, Chiba 271‐8510 Japan; ^5^ Cluster of Excellence on Plant Sciences (CEPLAS) University of Cologne, Botanical Institute Cologne Germany

## Abstract

Plant pathogens secrete effector molecules during host invasion to promote colonization. However, some of these effectors become recognized by host receptors to mount a defence response and establish immunity*.* Recently, a novel resistance was identified in wild tomato, mediated by the single dominant *V2* locus, to control *strains of* the soil‐borne vascular wilt fungus *Verticillium dahliae that belong to race 2*. With comparative genomics of race 2 strains and resistance‐breaking race 3 strains, we identified the avirulence effector that activates *V2* resistance, termed Av2*.* We identified 277 kb of race 2‐specific sequence comprising only two genes encoding predicted secreted proteins that are expressed during tomato colonization. Subsequent functional analysis based on genetic complementation into race 3 isolates and targeted deletion from the race 1 isolate JR2 and race 2 isolate TO22 confirmed that one of the two candidates encodes the avirulence effector Av2 that is recognized in *V2* tomato plants. Two *Av2* allelic variants were identified that encode Av2 variants that differ by a single acid. Thus far, a role in virulence could not be demonstrated for either of the two variants.

## Introduction

In nature, plants are continuously threatened by potential plant pathogens. However, most plants are resistant to most potential plant pathogens due to an efficient immune system that becomes activated by any type of molecular pattern that accurately betrays microbial invasion (Dangl and Jones, 2001; Cook *et al*., 2015). Throughout time, different conceptual frameworks have been put forward to describe the molecular basis of plant–pathogen interactions and the mechanistic underpinning of plant immunity. Initially, Harold Flor introduced the gene‐for‐gene model in which a single dominant host gene, termed a resistance (*R*) gene, induces resistance in response to a pathogen expressing a single dominant avirulence (*Avr*) gene (Flor, 1942). Isolates of the pathogen that do not express the allele of the *Avr* gene that is recognized escape recognition and are assigned to a resistance‐breaking race. In parallel to these race‐specific Avrs, non‐race‐specific elicitors were described as conserved microbial molecules that are often recognized by multiple plant species (Darvill and Albersheim, 1984). The recognition by plants of Avrs and of non‐race‐specific elicitors, presently known as pathogen‐ or microbe‐associated molecular patterns (P/MAMPs), was combined in the ‘zig‐zag’ model (Jones and Dangl, 2006). In this model, P/MAMPs are perceived by cell surface‐localized pattern recognition receptors (PRRs) to trigger pattern‐triggered immunity (PTI), while effectors are recognized by cytoplasmic receptors that are known as resistance (R) proteins to activate effector‐triggered immunity (ETI) (Jones and Dangl, 2006). Importantly, the model recognizes that Avrs function to suppress host immune responses in the first place, implying that these molecules, besides being avirulence determinants, act as virulence factors through their function as effector molecules (Jones and Dangl, 2006). A more recent model, termed the invasion model, recognizes that the functional separation of PTI and ETI is problematic and proposes that the corresponding receptors, collectively termed invasion pattern receptors (IPRs), detect either externally encoded or self‐modified ligands that indicate invasion, termed invasion patterns (IPs), to mount an effective immune response (Thomma *et al*., 2011; Cook *et al*., 2015). However, it is generally appreciated that microbial pathogens secrete dozens to hundreds of effectors to contribute to disease establishment, only some of which are recognized as Avrs (Rovenich *et al*., 2014).

IPRs encompass typical *R* genes, which have been exploited for almost a century to confer resistance against plant pathogens upon introgression from sexually compatible wild relatives into elite cultivars (Dodds and Rathjen, 2010; Dangl *et al*., 2013). Most *R* genes encode members of a highly polymorphic superfamily of intracellular nucleotide‐binding leucine‐rich repeat (NLR) receptors, while others encode cell surface receptors (Dangl *et al*., 2013). Unfortunately, most *R* genes used in commercial crops are short‐lived because the resistance that they provide is rapidly broken by pathogen populations as their deployment in monoculture‐based cropping systems selects for pathogen variants that overcome immunity (Stukenbrock and McDonald, 2008; Dangl *et al*., 2013). Such breaking of resistance occurs upon purging of the *Avr* gene, sequence diversification, or by employment of novel effectors that subvert the host immune response (Stergiopoulos *et al*., 2007; Cook *et al*., 2015).

The molecular cloning of the first bacterial *Avr* gene from *Pseudomonas syringae* pv. *glycinea* was reported in 1984 (Staskawicz *et al*., 1984), the first fungal *Avr* gene from *Cladosporium fulvum* in 1991 (van Kan *et al*., 1991) and the first oomycete *Avr* gene from *Phytophthora sojae* in 2004 (Shan *et al*., 2004). Dozens of additional *Avr* genes have been cloned since then, in various pathogens (Parlange *et al*., 2009; Inami *et al*., 2012; Plissonneau *et al*., 2016; Niu *et al*., 2016; Lu *et al*., 2016; Zhong *et al*., 2017; Salcedo *et al*., 2017; Praz *et al*., 2016; Inoue *et al*., 2017; Chen *et al*., 2017; Meile *et al*., 2018; Kema *et al*., 2018; Anh *et al*., 2018; Bourras *et al*., 2015, 2019; Petit‐Houdenot *et al*., 2019; Saur *et al*., 2019). Most of these *Avr* genes have been identified by map‐based cloning and reverse genetics strategies. More recently, advances in (the affordability of) genome sequencing have allowed the cloning of novel *Avrs* by combining comparative genomics or transcriptomics with functional assays, a trend that was spearheaded by the cloning of the first *Avr* gene from *Verticillium dahliae* only in 2012 (de Jonge *et al*., 2012; Mesarich *et al*., 2014; Schmidt *et al*., 2016).

*Verticillium dahliae* is a soil‐borne fungal pathogen and causal agent of Verticillium wilt on a broad range of host plants that comprises hundreds of dicotyledonous plant species, including numerous crops such as tomato, potato, lettuce, olive, and cotton (Fradin and Thomma, 2006; Klosterman *et al*., 2009). The first source of genetic resistance toward *Verticillium* wilt was identified in tomato (*Solanum lycopersicum*) in the early 1930s in an accession called Peru Wild (Schaible *et al*., 1951). The resistance is governed by a single dominant locus, designated *Ve* (Diwan *et al*., 1999), comprising two genes that encode cell surface receptors of which one, *Ve1*, acts as a genuine resistance gene (Fradin and Thomma, 2006). Shortly after its deployment in the 1950s, resistance‐breaking strains have appeared that were assigned to race 2, whereas strains that are contained by *Ve1* belong to race 1 (Alexander, 1962). Thus, *Ve1* is characterized as a race‐specific *R* gene, and resistance‐breaking strains have become increasingly problematic over time (Alexander, 1962; Dobinson *et al*., 1996). With comparative population genomics of race 1 and race 2 strains, the *V. dahliae* avirulence effector that is recognized by tomato Ve1 was identified as VdAve1, an effector that is secreted during host colonization (de Jonge *et al*., 2012). As anticipated, it was demonstrated that VdAve1 acts as a virulence factor on tomato plants that lack the *Ve1* gene and that, consequently, cannot recognize VdAve1 (de Jonge *et al*., 2012). Recent evidence demonstrates that VdAve1 exerts selective antimicrobial activity and has the capacity to manipulate local microbiomes inside host plants as well as in the environment (Snelders *et al*., 2020). Whereas all race 1 strains carry an identical copy of *VdAve1*, all race 2 strains analysed to date are characterized by complete loss of the *VdAve1* locus (de Jonge *et al*., 2012; Faino *et al*., 2016). Intriguingly, phylogenetic analysis has revealed that *VdAve1* was horizontally acquired by *V. dahliae* from plants (de Jonge *et al*., 2012; Shi‐Kunne *et al*., 2018), after which the effector gene was lost multiple times independently, presumably due to selection pressure exerted by the *Ve1* locus that has been introgressed into most tomato cultivars (Faino *et al*., 2016).

Despite significant efforts, attempts to identify genetic sources for race 2 resistance in tomato have remained unsuccessful for a long time (Baergen *et al*., 1993). Recently, however, a source of race 2 resistance was identified in the wild tomato species *Solanum neorickii* (Usami *et al*., 2017). This genetic material was used to develop the tomato rootstock cultivars Aibou, Ganbarune‐Karis and Back Attack by Japanese breeding companies, in which resistance is controlled by a single dominant locus, denoted *V2* (Usami *et al*., 2017). However, experimental trials using race 2‐resistant rootstocks revealed resistance‐breaking *V. dahliae* strains that, consequently, are assigned to race 3 (Usami *et al*., 2017). In this study, we performed comparative genomics combined with functional assays to identify the avirulence effector Av2 that activates race‐specific resistance in tomato genotypes that carry *V2*.

## Results

### Identification of *Verticillium dahliae* strains that escape *V2* resistance

**Fig. 1:**
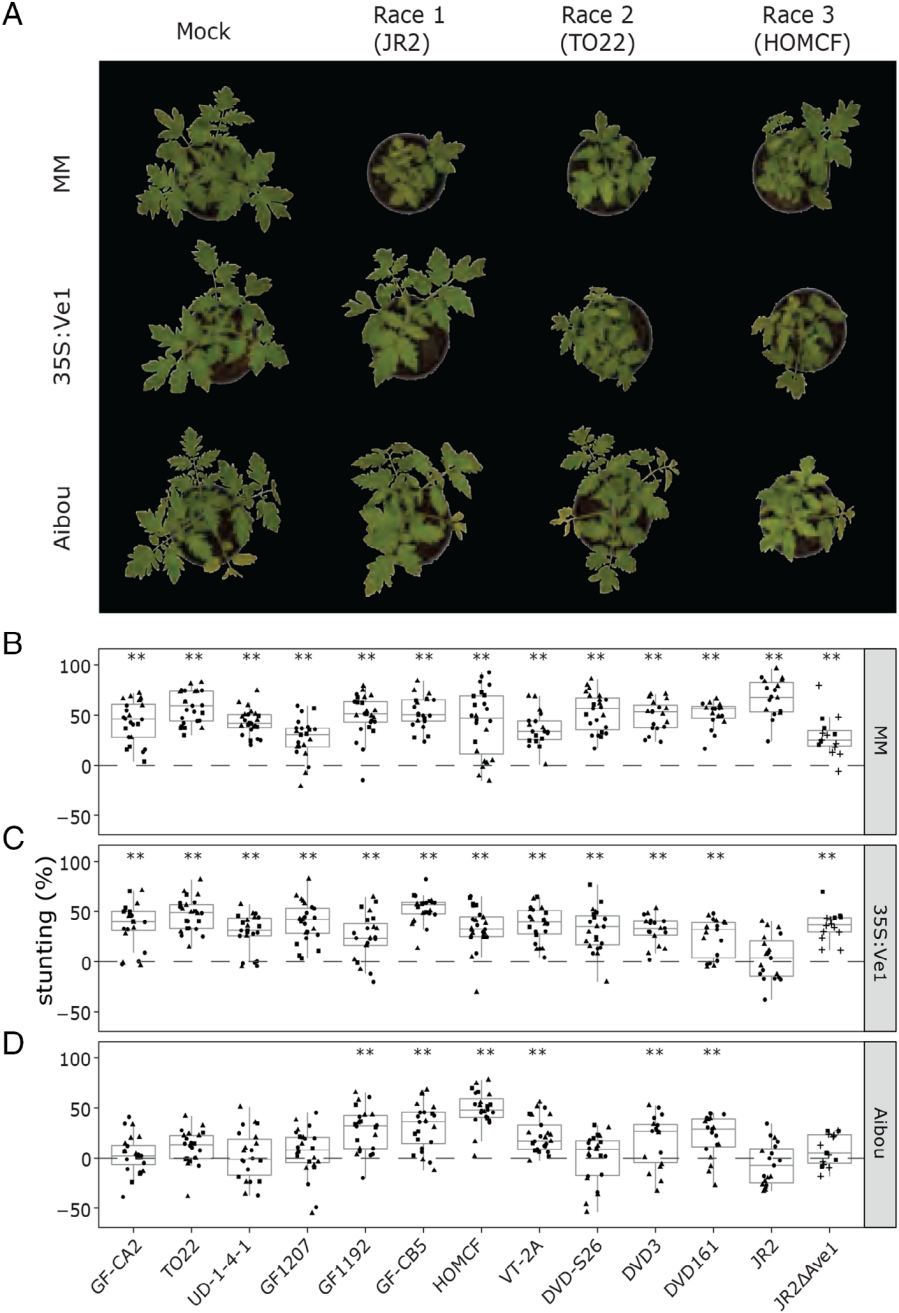
Pathogenicity phenotyping of a collection of *Verticillium dahliae* strains on tomato. A. Typical appearance of *V dahliae* infection by strain JR2, TO22 and HOMCF as representatives for race 1, 2 and 3, respectively, on Moneymaker (MM) plants that lack known *V. dahliae* resistance genes, *Ve1*‐transgenic Moneymaker plants that are resistant against race 1 and not against race 2 or 3 strains, and Aibou plants that carry *Ve1* and *V2* and are therefore resistant against race 1 as well as race 2 strains, but not against race 3 strains at 21 days post inoculation (dpi). B–D. Measurement of *V. dahliae*‐induced stunting on wild‐type Moneymaker plants (B), *Ve1*‐transgenic Moneymaker plants (35S:Ve1) (C) and Aibou plants (D) at 21 dpi. The graphs show collective data from four different experiments indicated with different symbols (circles, squares, triangles and plus symbols), and asterisks indicate significant differences between *V. dahliae*‐ and mock‐inoculated plants as determined with an ANOVA followed by a Fisher's LSD test (*P* < 0.01). [Color figure can be viewed at wileyonlinelibrary.com]

To identify *Av2* as the *V. dahliae* gene that mediates **a**virulence on tomato ***V2*** plants, we pursued a comparative genomics strategy by searching for genomic regions that are absent from all race 3 strains. To this end, we performed pathogenicity assays with a collection of *V. dahliae* strains on a differential set of tomato genotypes, comprising (I) Moneymaker plants that lack *V. dahliae* resistance genes, (II) *Ve1*‐transgenic Moneymaker plants that are resistant against race 1 and not against race 2 strains (Fradin *et al*., 2009), and (III) Aibou plants that carry *Ve1* and *V2* and are therefore resistant against race 1 as well as race 2 strains (Usami *et al*., 2017) (Fig. 1A). First, we aimed to confirm the race assignment of eight *V. dahliae* strains that were previously tested by Usami *et al*. (2017) (Table 1). Additionally, three strains that were previously assigned to race 2 were included (de Jonge *et al*., 2012) as well as *V. dahliae* strain JR2 (race 1) because of its gapless telomere‐to‐telomere assembly (Faino *et al*., 2015).

**Table 1:** *Verticillium dahliae* strains used in this study for comparative genomics and genome assembly statistics.

Strain	Previous race annotation	Ref. isolate^a^	Novel race annotation	Platform^b^	Data (Gb)^c^	Assembly size (Mb)	No. of contigs^d^	Contig N50 (Kb) ^4^	Ref. sequencing^a^
JR2	1	A	1	PacBio	8.9	36.1	8	4168	D
DVDS26	2	B	2	Illumina	1	35.3	5361	47.1	B
DVD161	2	B	2, 3	Illumina	1	34.1	4078	42.4	B
DVD3	2	B	2, 3	Illumina	1	34.1	9318	43.9	B
TO22	2	C	2	Nanopore	4	34.9	20	12.4	TS
UD1‐4‐1	2	C	2	Nanopore	1.9	34.6	18	18.1	TS
GF1207	2	C	2	Nanopore	1.6	34.8	69	8.5	TS
GF‐CA2	2	C	2	Nanopore	2	35	38	9.1	TS
GF‐CB5	3	C	2, 3	Nanopore	4	34.8	19	11.4	TS
VT‐2A	3	C	2, 3	Nanopore	1.8	34.8	22	10.2	TS
GF1192	3	C	2, 3	Nanopore	2	34.6	23	14.5	TS
HOMCF	3	C	2, 3	Nanopore	2	36.1	33	10.1	TS

^a^ References: A: Fradin *et al*., 2009; B: de Jonge *et al*., 2012; C: Usami *et al*., 2017; D: Faino *et al*., 2015; TS: this study.

^b^ Sequencing platform used.

^c^ Amount of sequencing data generated.

^d^ For strains DVDS26, DVD161 and DVD3 previously determined scaffold statistics are shown (de Jonge *et al*., 2012).

At 3 weeks post inoculation, all strains caused significant stunting on the universally susceptible Moneymaker control (Fig. 1A and B), while all strains except for the race 1 strain JR2 caused significant stunting on *Ve1*‐transgenic Moneymaker plants (Fig. 1A and C), corroborating that, except for strain JR2, none of the strains belongs to race 1 and that a potential containment on Aibou plants cannot be caused by Ve1 recognition of the VdAve1 effector. Importantly, all of the strains that were used by Usami *et al*. (2017) and that were previously assigned to race 2 did not cause significant stunting on Aibou, whereas all of the strains that were assigned to race 3 caused clear symptoms of Verticillium wilt disease (Fig. 1, Table 1; Usami *et al*., 2017). The previously assigned race 2 strain DVDS26 (de Jonge *et al*., 2012) caused no significant stunting on Aibou plants, confirming that this remains a race 2 strain, while strains DVD161 and DVD3 caused significant stunting, implying that these strains should actually be assigned to race 3. As expected, the race 1 strain JR2 did not cause stunting on Aibou plants, which can at least partially be attributed to VdAve1 effector recognition by the *Ve1* gene product in these plants. However, the finding that a transgenic *VdAve1* deletion line (JR2Δ*Ave1*; de Jonge *et al*., 2012) caused significant stunting on *Ve1*‐transgenic Moneymaker and not on Aibou plants, indicates that the JR2 strain might also encode Av2. Currently, it is not known whether this is the case, or whether it is simply that basal defence is enhanced in the absence of *Ave1*. After all, we previously showed that the virulence of the *VdAve1* deletion strain on tomato is severely compromised (de Jonge *et al*., 2012), which can also be observed on Moneymaker plants in our assays (Fig. 1B). This observation, combined with the observation that stunting on Aibou plants by any race 3 strain is generally less than stunting on Moneymaker plants (Fig. 1B and D), could indicate that basal defence against Verticillium wilt is enhanced in Aibou plants, and thus that incompatibility of the *VdAve1* deletion strain may be due to enhanced basal defence rather than due to V2‐mediated recognition of the JR2 strain.

### Comparative genomics identifies *Verticillium dahliae Av2* candidates

Besides the gapless genome assembly of strain JR2 (Faino *et al*., 2015), genome assemblies were also available for strains DVDS26, DVD161 and DVD3, albeit that these assemblies were highly fragmented as these were based on Illumina short‐read sequencing data (de Jonge *et al*., 2012) (Table 1). In this study, we determined the genomic sequences of the race 2 strains TO22, UD1‐4‐1, GF1207 and GFCA2, and the race 3 strains GF‐CB5, GF1192, VT2A and HOMCF with Oxford Nanopore sequencing Technology (ONT) using a MinION device (Table 1). For each strain, ~2–4 Gb of sequence data was produced, representing 50–100x genome coverage based on the ~35 Mb gapless reference genome of *V. dahliae* strain JR2 (Faino *et al*., 2015). Subsequently, we performed self‐correction of the reads, read trimming and genome assembly, leading to genome assemblies ranging from 18 contigs for strain UD1‐4‐1 to 69 for strain GF1207 (Table 1).

Based on the genome sequences, we pursued comparative genomics analyses by exploring two scenarios. The first scenario is that *Av2* is race 2‐specific and thus present in race 2 lineage sequences while absent from race 3. The second scenario is that *Av2* is present in isolates that belong to race 1 and race 2, but that the resistance phenotype against race 2 is masked by Ve1 resistance directed against Ave1. In scenario I, comparative genomics was performed making use of race 2 strain TO22 (Usami *et al*., 2017) as a reference, while in scenario II race 1 strain JR2 (Faino *et al*., 2015) was used (Table 2). To this end, self‐corrected reads from the *V. dahliae* race 3 strains were mapped against the assembly of *V. dahliae* strain TO22 (scenario I) or strain JR2 (scenario II) and regions that were not covered by race 3 reads were retained (Table 2). Next, self‐corrected reads from the race 2 strains were mapped against the retained reference genome‐specific regions that are absent from the race 3 strains, and sequences that were found in every race 2 strain were retained as candidate regions to encode the Avr molecule. Sequences that are shared by the *V. dahliae* strain TO22 reference assembly and all race 2 strains, and that are absent from all race 3 strains, were mapped against the *V. dahliae* strain JR2 genome assembly, and common genes were extracted. Sequences that did not map to the *V. dahliae* strain JR2 genome assembly were *de novo* annotated and signal peptides for secretion at the N‐termini of the encoded proteins were predicted to identify potential effector genes.

**Table 2:** Comparative genomics of race 2 and race 3 strains.

Scenario	I	II
Reference strain	TO22	JR2
Race 2	GF‐CA2 TO22 UD‐1‐4‐1 DVDS26 GF1207	GF‐CA2 TO22 UD‐1‐4‐1 DVDS26 GF1207
Race 3	GF‐1192 GF‐CB5 HOMCF DVD161 DVD3 VT‐2A	GF‐1192 GF‐CB5 HOMCF DVD161 DVD3 VT‐2A
Retained (kb)	563	222
Shared with JR2 (kb)	222	222
#JR2 genes	40	40
#Augustus‐predicted genes	70	‐‐
#Secreted	6	2
Retained candidates	XLOC_00170	XLOC_00170
	evm.model.contig1569.344	evm.model.contig1569.344
	tig00000058:1 027 588–1 028 906	
	tig00000058:1 116 044–1 116 494	
	tig00000151:403362–404 089	
	tig00017428:835657–837 290	

Our strategy identified 563 kb of race 2‐specific regions, containing 110 genes of which six encode putative secreted proteins, for scenario I (Table 2). For approach II, 222 kb of sequence that lacks in race 3 strains was identified with 40 genes of which only two are predicted to encode secreted proteins; *XLOC_00170* (*VDAG_JR2_Chr4g03680a*) and *evm.model.contig1569.344* (*VDAG_JR2_Chr4g03650a*, further referred to as *Evm_344*). Intriguingly, both these genes were previously recognized as being among the most highly expressed effector genes during colonization of *Nicotiana benthamiana* plants (de Jonge *et al*. 2013; Faino *et al*., 2015).

### Only two of the *Av2* candidates are expressed *in planta*

**Fig. 2:**
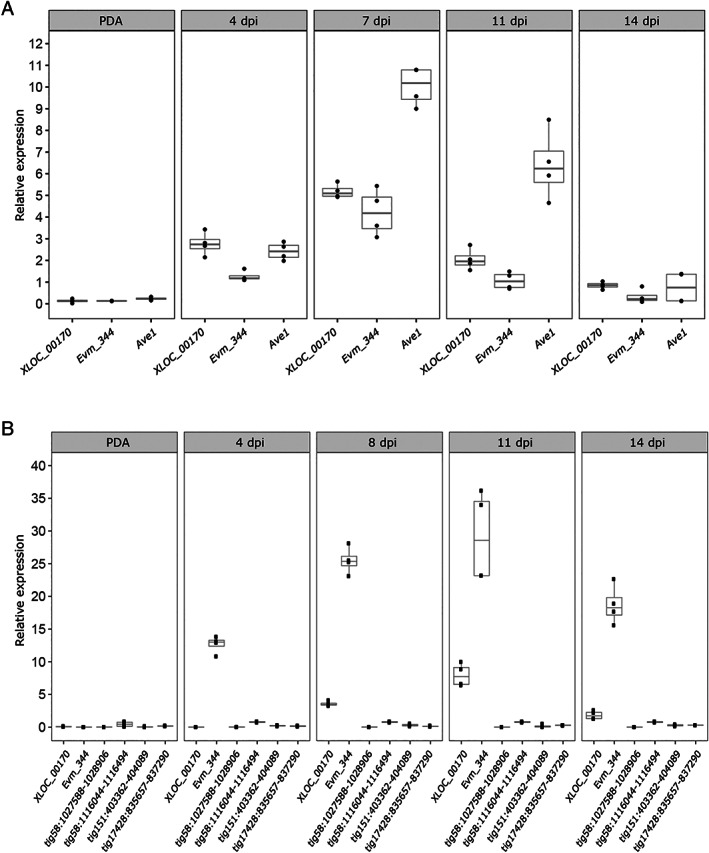
Expression of *V. dahliae* candidate avirulence effector genes *in vitro* and during colonization of tomato plants. To assess *in planta* expression, 12‐day‐old tomato cv. Moneymaker seedlings were root‐inoculated with *V. dahliae* strain JR2 (A) or strain TO22 (B), and plants were harvested from 4 to 14 days post inoculation (dpi), while conidiospores were harvested from 5‐day‐old cultures of *V. dahliae* on potato dextrose agar (PDA) to monitor *in vitro* expression. Real‐time PCR was performed to determine the relative expression of *XLOC_00170, Evm_344* and the race 1‐specific effector gene *VdAve1* as a positive control (de Jonge *et al*., 2012) for strain JR2, using *V. dahliae GAPDH* as reference (A). Similarly, the relative expression of *XLOC_00170, Evm_344* and six additional avirulence effector genes for strain TO22, using *V. dahliae GAPDH* as reference (B).

We anticipate that the genuine *Av2* gene may not necessarily be expressed in *N. benthamiana* (de Jonge *et al*. 2013) but should be expressed particularly in tomato. Real‐time PCR analysis on a time course of tomato cultivar Moneymaker plants inoculated with the *V. dahliae* JR2 strain revealed that the two candidate genes are expressed during tomato colonization, with a peak in expression around 7 days post inoculation, whereas little to no expression could be recorded upon growth *in vitro* (Fig. 2A). Both genes are similarly expressed in *V. dahliae* strain TO22, albeit that the expression peaks slightly later, at 11 dpi (Fig. 2B). However, whereas the expression level of both genes is similar in *V. dahliae* strain JR2, *Evm_344* is higher expressed than *XLOC_00170* in *V. dahliae* strain TO22. Importantly, none of the four additional avirulence effector gene candidates that were identified in comparative genomics scenario I is expressed *in planta* in *V. dahliae* strain TO22 (Fig. 2B). Thus, based on the transcriptional profiling, these four avirulence effector genes can be disqualified as *Av2* candidates, and only two genes that display an expression profile that can be expected for a potential avirulence effector gene remain; *XLOC_00170* and *Evm_344*.

### *XLOC_00170* encodes Av2

**Fig. 3:**
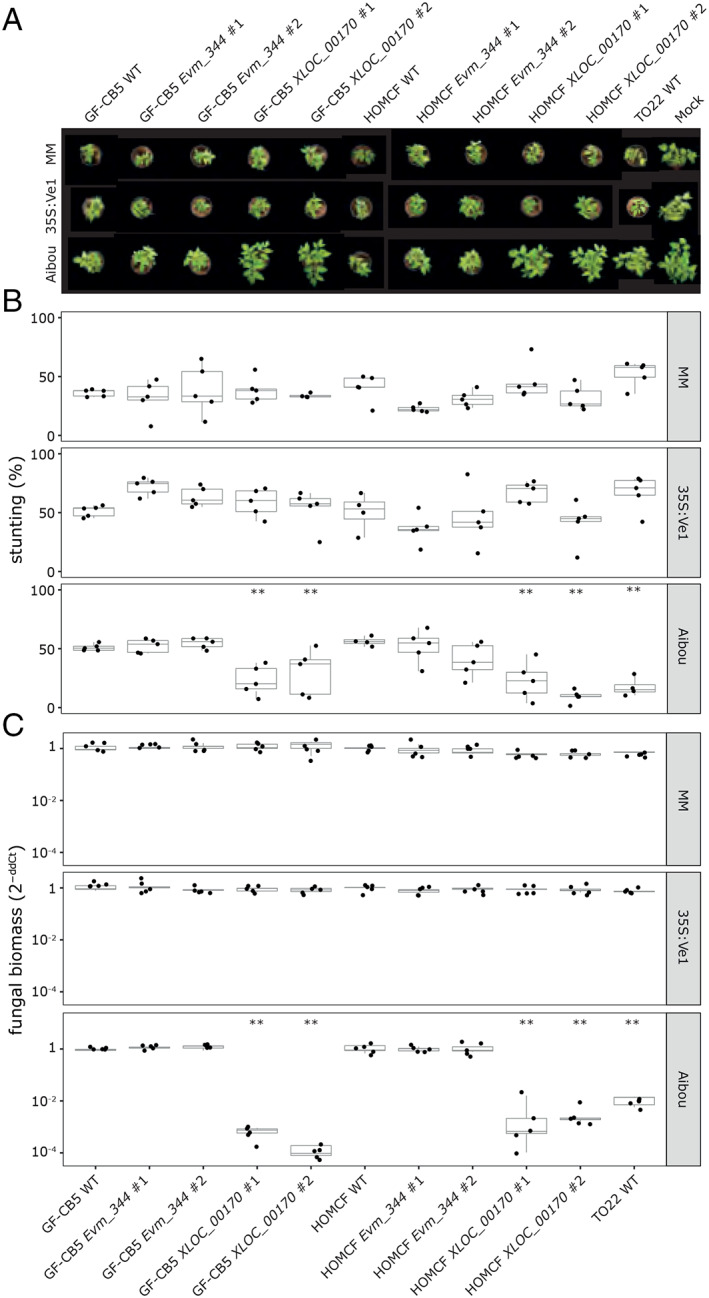
Genetic complementation demonstrates that *XLOC_00170* encodes the avirulence effector Av2 that is recognized in *V2* plants. A. Top pictures of Moneymaker plants that lack known *V. dahliae* resistance genes (MM), *Ve1*‐transgenic Moneymaker plants that are resistant against race 1 and not against race 2 strains of *V. dahliae* (MM 35S:Ve1), and Aibou plants that carry *Ve1* and *V2* and are therefore resistant against race 1 as well as race 2 strains of the pathogen (Usami *et al*., 2017) inoculated with the race 3 WT strains GF‐CB5 and HOMCF, and two independent genetic complementation lines that express *XLOC_00170* or *Evm_344*, and the race 2 strain TO22. B. Quantification of stunting caused by the various *V. dahliae* genotypes on the various tomato genotypes as detailed for panel (A). Each combination is represented by the measurement of five plants. C. Quantification of fungal biomass with real‐time PCR determined for the various *V. dahliae* genotypes on the various tomato genotypes as detailed for panel (A). Each combination is represented by the fungal biomass quantification in five plants. Asterisks indicate significant differences between *V. dahliae*‐ and mock‐inoculated plants as determined with an ANOVA followed by a Fisher's LSD test (*P* < 0.01). [Color figure can be viewed at wileyonlinelibrary.com]

To identify which of the two candidates encodes Av2, a genetic complementation approach was pursued in which the two candidate genes were introduced individually into the *V. dahliae* race 3 strains GF‐CB5 and HOMCF. Subsequently, inoculations were performed on a differential set of tomato genotypes, comprising Moneymaker plants, *Ve1*‐transgenic Moneymaker plants (Fradin *et al*., 2009), and Aibou plants (Usami *et al*., 2017). As expected, the non‐transformed race 3 strains GF‐CB5 and HOMCF as well as the complementation lines containing *XLOC_00170* or *Evm_344* caused clear stunting of the universally susceptible Moneymaker as well as of the *Ve1‐*transgenic Moneymaker plants (Fig. 3A and B). Interestingly, non‐transformed race 3 strains GF‐CB5 and HOMCF and the *Evm_344* complementation lines caused clear stunting on Aibou plants, whereas the *XLOC_00170* complementation lines did not induce disease symptoms and stunting on these plants (Fig. 3A and B). As such, these complementation transformants of the race 3 strains GF‐CB5 and HOMCF behaved essentially as the race 2 strain TO22 (Fig. 3A and B). Thus, these findings suggest that *XLOC_00170* encodes Av2. All visual observations of stunting were supported by quantifications of fungal biomass by real‐time PCR (Fig. 3C). These measurements revealed that fungal biomass levels were only reduced on Aibou plants when inoculated with the race 2 strain TO22, and with the race 3 strains GF‐CB5 and HOMCF that were complemented with *XLOC_00170*. Thus, our data confirm that reduced symptomatology is accompanied by significantly reduced fungal colonization and indicate that *XLOC_00170* encodes the race 2‐specific avirulence effector Av2.

**Fig. 4:**
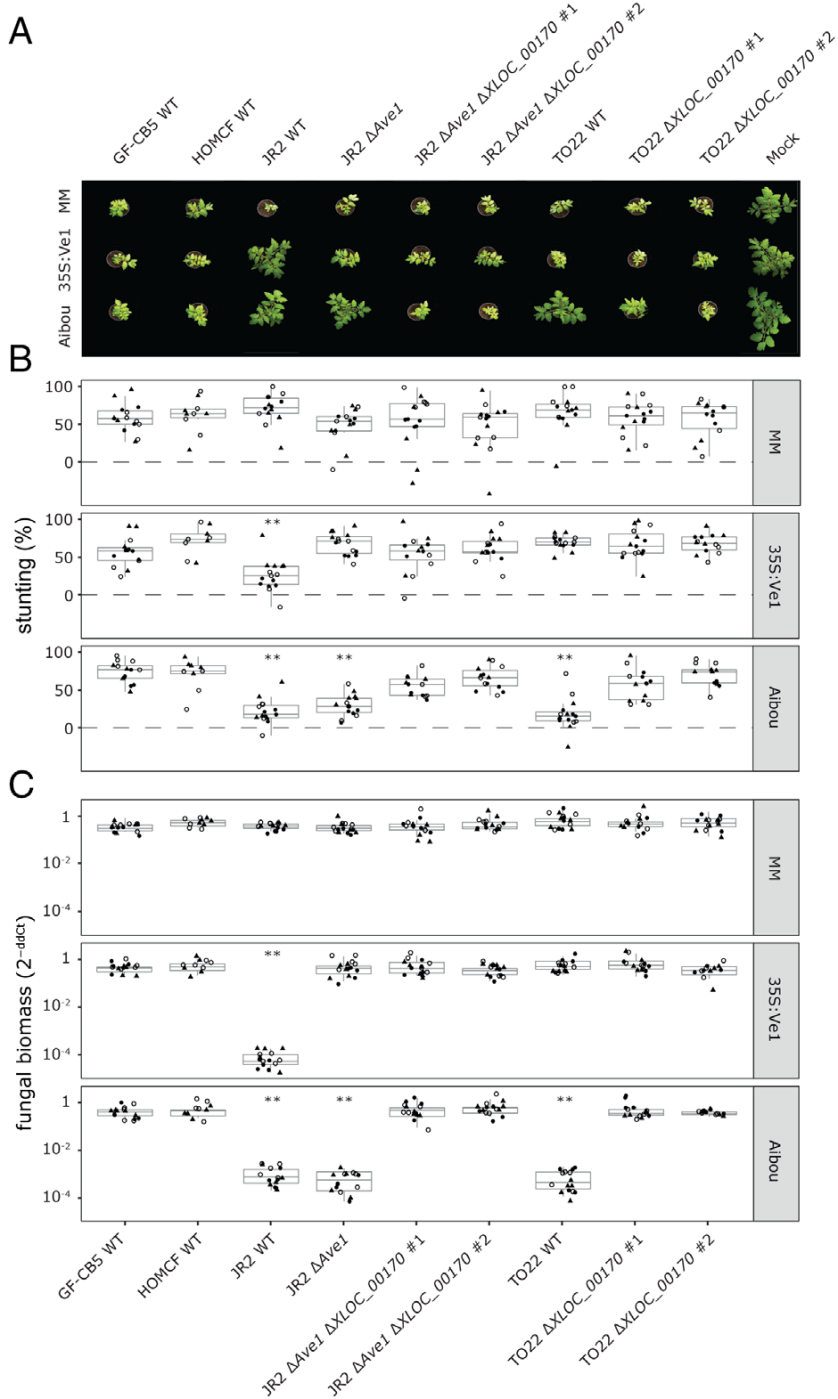
Targeted deletion confirms that *XLOC_00170* encodes the avirulence effector Av2 that is recognized in *V2* plants. A. Top pictures of Moneymaker plants that lack known *V. dahliae* resistance genes (MM), *Ve1*‐transgenic Moneymaker plants that are resistant against race 1 and not against race 2 strains of *V. dahliae* (35S:Ve1), and Aibou plants that carry *Ve1* and *V2* and are therefore resistant against race 1 as well as race 2 strains of the pathogen (Usami *et al*., 2017) inoculated with the race 3 strains GF‐CB5 and HOMCF, the race 1 WT strain JR2, the deletion line JR2Δ*Ave1*, two independent knock‐out lines of *XLOC_00170* in JR2Δ*Ave1*, the race 2 WT strain TO22 and two independent knock‐out lines of *XLOC_00170* in TO22. B. Quantification of stunting. C. Quantification of fungal biomass with real‐time PCR caused by the various *V. dahliae* genotypes on the various tomato genotypes as detailed for panel (A). Different symbols (empty circles, filled circles and triangles) refer to five plants from three different experiments. Asterisks indicate significant differences between *V. dahliae*‐ and mock‐inoculated plants as determined with an ANOVA followed by a Fisher's LSD test (*P* < 0.01). [Color figure can be viewed at wileyonlinelibrary.com]

To further confirm that *XLOC_00170* encodes Av2, targeted gene deletions were pursued in race 2 strain TO22 as well as in the JR2Δ*Ave1* strain and inoculations were performed on Moneymaker plants, Ve1‐transgenic Moneymaker plants (Fradin *et al*., 2009), and Aibou plants (Usami *et al*., 2017). All *V. dahliae* genotypes caused clear stunting on wild‐type and *Ve1*‐transgenic Moneymaker plants, except for wild‐type JR2 on *Ve1*‐transgenic Moneymaker (Fig. 4A and B). Interestingly, whereas *V. dahliae* strains TO22 and JR2Δ*Ave1* were contained on Aibou plants, the *XLOC_00170* deletion strains caused stunting of these plants in a similar fashion as the race 3 strains GF‐CB5 and HOMCF (Fig. 4C). All visual observations were supported by quantification of biomass by real‐time PCR (Fig. 4C). Collectively, our data unambiguously demonstrate that *XLOC_00170* encodes the Av2 effector that is recognized on *V2* tomato plants.

### *Av2* does not seem to contribute to virulence

It has been widely recognized that the intrinsic function of Avrs is to support host colonization by acting as virulence determinants (Jones and Dangl, 2006; Rovenich *et al*., 2014; Cook *et al*., 2015). Thus, we assessed the virulence of the complementation lines alongside their wild‐type progenitor genotypes on wild‐type and *Ve1*‐transgenic Moneymaker plants (Fig. 3). However, no significant increase in symptomatology nor in fungal colonization could be recorded upon *Av2* introduction. Similarly, no significant decrease in symptomatology, nor a decrease in fungal colonization could be recorded upon *Av2* deletion from *V. dahliae* strain TO22 on these tomato genotypes and upon *Av2* deletion from JR2Δ*Ave1* on wild‐type Moneymaker plants (Fig. 4), suggesting that Av2 is not a major contributor to virulence on tomato under the conditions of our assays.

### *Av2* distribution and allelic variation

**Fig. 5:**
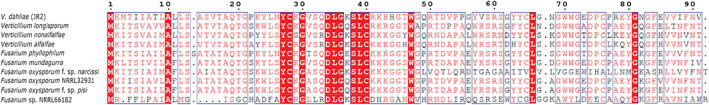
Amino acid alignment of Av2 homologues found in few fungal species. Based on BLAST analyses, homologues of *V. dahliae* Av2 could only be identified in *V. longisporum*, *V. nonalfalfae*, *V. alfalfa* and in *Fusarium phyllophilum*, *F. mundagurra*, three *F. oxysporum* lineages and in *Fusarium* sp. NRRL66182. Global alignments were performed using ClustalW and visualized with Espript3. Conserved amino acids are shown in white font on red background. [Color figure can be viewed at wileyonlinelibrary.com]

*Av2* encodes a 91 amino acid protein that, after removal of a predicted signal peptide, leaves a mature protein of 73 amino acids that includes four cysteine residues and that lacks known protein domains. Intriguingly, an Av2 homologue is found in *V. nonalfalfae* (78% identity), *V. longisporum* (68% identify) and *V. alfalfae* (49%) that, like *V. dahliae*, belong to the Flavnonexudans clade of *Verticillium* spp. (Fig. 5; Shi‐Kunne *et al*., 2018). Furthermore, BLAST searches revealed homologues in *Fusarium phyllophilum* (79% identity), *Fusarium mundagurra* (78%), *F. oxysporum* f. sp. *narcissi* (77%), *Fusarium oxysporum* NRRL32931 (75%), *F. oxysporum* f. sp. *pisi* (73%) and *Fusarium* sp. NRRL66182 (41%) (Fig. 5). No homologues are found in any of the other *Fusarium* spp nor in any other species.

**Fig. 6:**
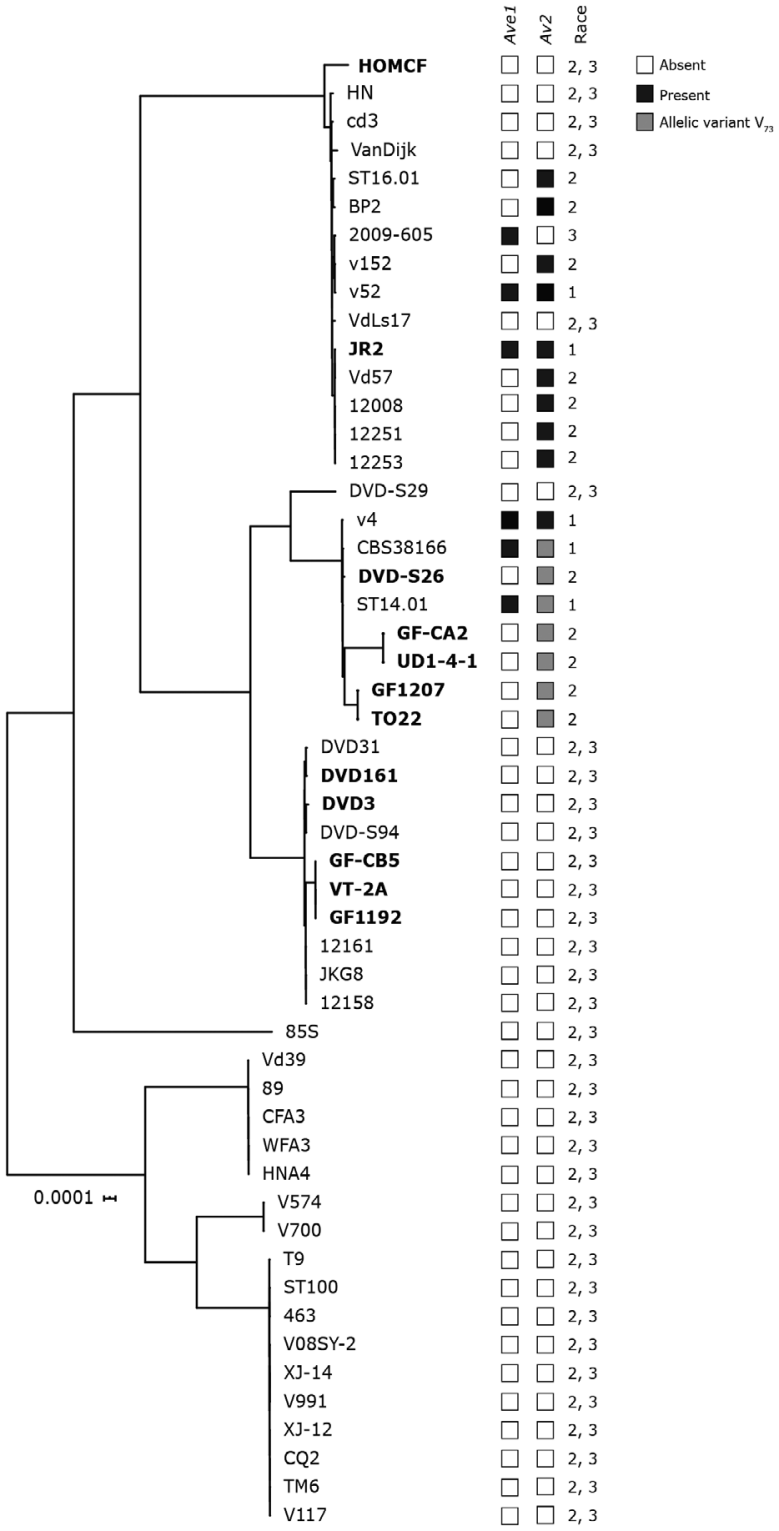
Phylogenetic tree of sequenced *V. dahliae* strains with indication of presence‐absence variation for the Ave1 and Av2 effectors. Strains that were phenotyped and included in the comparative genomics (Table 2) are shown in bold. Presence of the avirulence genes *VdAve1* and *Av2*, and the race designation based on the presence or absence of these genes are indicated. Phylogenetic relationships between sequenced *V. dahliae* strains were inferred using Realphy (Langmead and Salzberg, 2012), and branch length represents sequence divergence.

To assess *Av2* distribution in *V. dahliae*, presence–absence variations (PAV) were assessed in a collection of 52 previously sequenced *V. dahliae* strains (Fig. 6; de Jonge *et al*., 2012; Faino *et al*., 2015; Fan *et al*., 2018; Gibriel *et al*., 2019), revealing that *Av2* occurred in 17 of the isolates including the four race 2 isolates that were sequenced in this study (Fig. 6). To assess the phylogenetic relationships between strains that carry *Av2*, a phylogenetic tree was generated, showing that the strains can be grouped into three major clades, two of which comprising strains that contain *Av2*. However, within these clades closely related strains occur that lost *Av2*, suggesting the occurrence of multiple independent losses (Fig. 6). Overall, no obvious phylogenetic structure is apparent with respect to effector presence within the *V. dahliae* population.

**Fig. 7:**
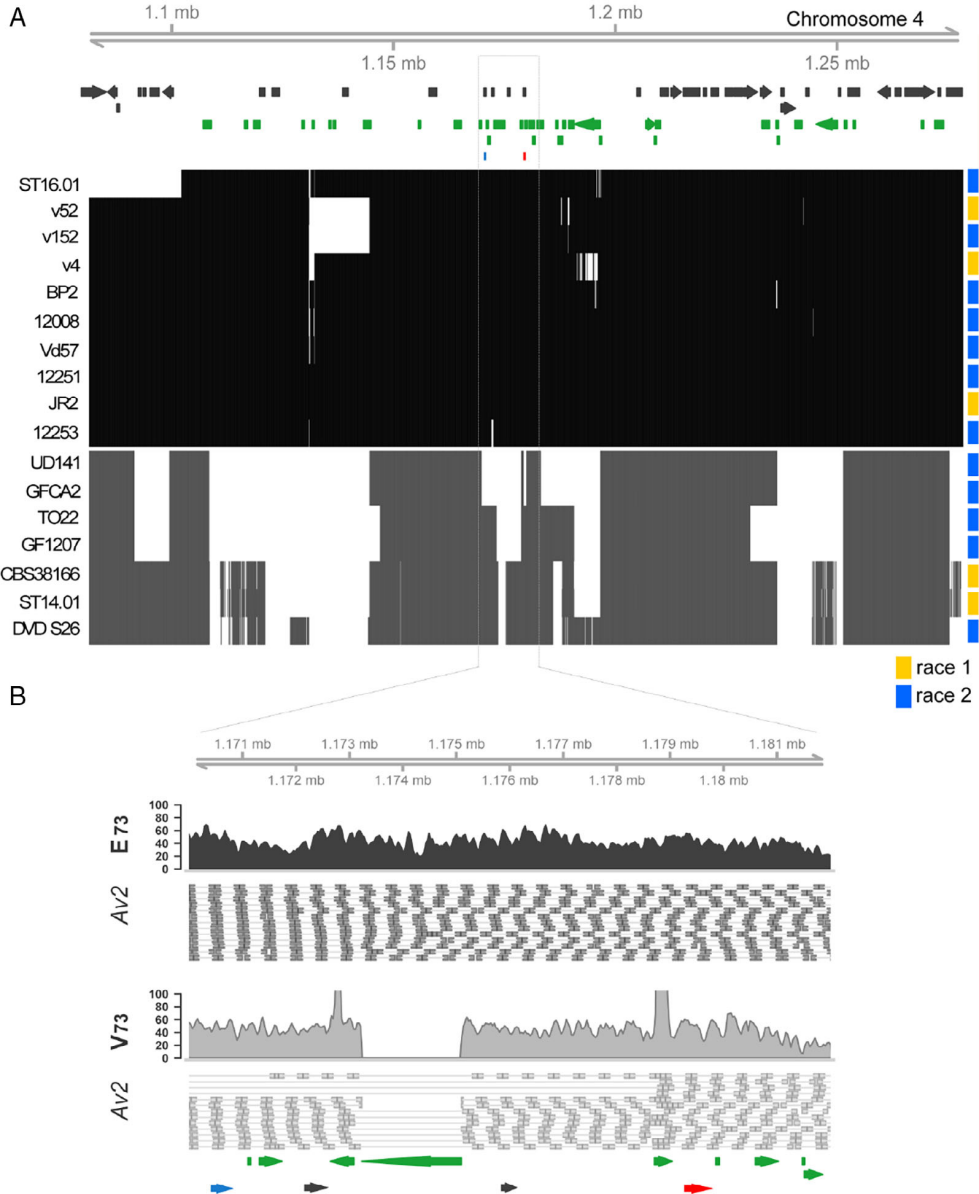
Presence–absence variation in the region surrounding the two candidate *Av2* genes (A) genomic region flanking the *Av2* candidate genes in 17 isolates detailed in Fig. 3. The matrix shows the presence (black/grey) and absence (white) in 100 bp non‐overlapping windows for *Av2* variant E_73_ (black) and *Av2* variant V_73_ grey. On top, annotated genes are displayed in black and repetitive elements in green, while *Av2* is displayed in red and *Evm_344* in blue. (B) Read coverage for *V. dahliae* strain JR2 that encodes *Av2* variant E_73_ and strain DVD‐S26 that encodes *Av2* variant V_73_ depicting a transposable element deletion in isolates that produce the V_73_ variant. [Color figure can be viewed at wileyonlinelibrary.com]

Next, we investigated the genomic organization surrounding *Av2* based on the gapless genome assembly of *V. dahliae* strain JR2 (Faino *et al*., 2015). Interestingly, *Av2* resides in close proximity to *Evm_344*, separated by only two additional genes, in a lineage‐specific (LS) region on chromosome 4 (Fig. 7). Furthermore, as typically observed in LS regions that are enriched in repetitive elements (de Jonge *et al*., 2013; Faino *et al*., 2016), *Av2* is surrounded by repetitive elements such as transposons that mostly belong to the class II long terminal repeat (LTR) retrotransposons (Fig. 7). Typically, LS regions are characterized by the high abundance of PAV. As expected, the flanking genomic regions (100 kb) are highly variable between *V. dahliae* strains (Fig. 7).

**Fig. 8:**
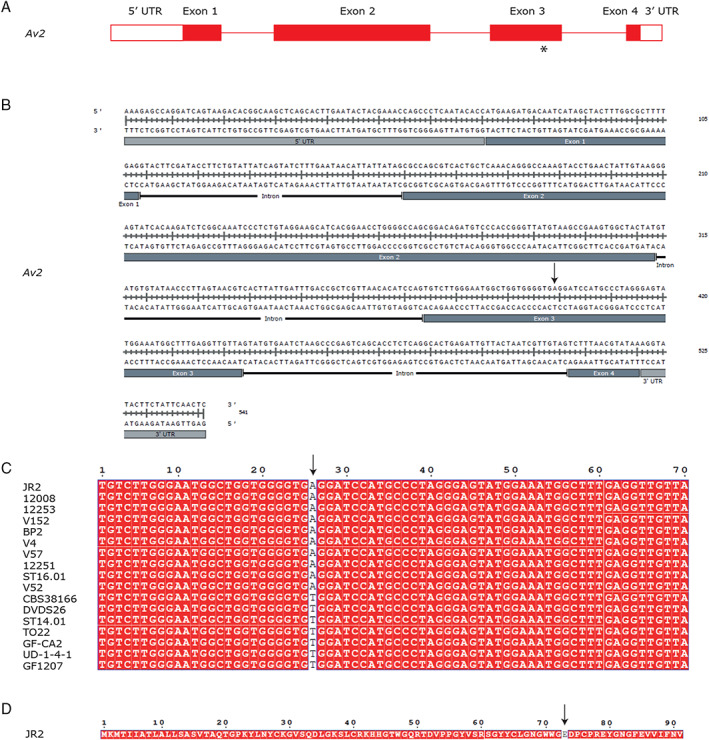
Allelic variation of *Av2* in *Verticillium dahliae*. A. Gene model for *Av2*. The asterisk indicates the approximate position of the single (A to T) nucleotide substitution in 7 of the 17 isolates that carry the gene, leading to a single amino acid substitution (E_73_V). B. Genomic sequence of *Av2*. The arrow shows the position of the single nucleotide substitution found in particular strains. C. Alignment of exon 3 of *Av2* in the 17 strains containing the avirulence effector gene. The arrow shows the single nucleotide substitution that occurs in seven of the strains when compared with strain JR2. D. Av2 amino acid sequence as encoded by *V. dahliae* strain JR2 with E_73_ that is substituted by V in seven isolates indicated by an arrow. [Color figure can be viewed at wileyonlinelibrary.com]

As many Avr effectors are under strong selection pressure and thus often display enhanced allelic variation (Stergiopoulos *et al*., 2007), we assessed allelic variation among the 17 *Av2* alleles identified in this study. We identified only two allelic variants within the 17 *Av2* alleles that differed by a single nucleotide polymorphism (SNP) in exon 3 leading to a polymorphic amino acid at position 73. Whereas 10 isolates carry a glutamic acid at this position (E_73_), seven other carry a valine (V_73_) (Fig. 8). Interestingly, strains carrying V_73_ are clustered in the same branch, suggesting that a single event caused this polymorphism (Fig. 6). We noticed that all isolates carrying E_73_ carry an extra transposable element of the DNA/Tc‐1 Mariner class in the upstream region of the *Av2* gene (Fig. 8). Intriguingly, as strains GF‐CA2, TO22, UD‐1‐4‐1 DVDS26 and GF1207, that encode the Av2 variant with V_73_, as well as JR2Δ*Ave1*, that encodes the variant with E_73_, are contained on Aibou plants, we conclude that both allelic variants are recognized by V2. Moreover, the *Av2* deletion strain of TO22 (with V_73_) as well as of JR2Δ*Ave1* (with E_73_) is not compromised in aggressiveness on wild‐type Moneymaker plants when compared with the TO22 or JR2Δ*Ave1* progenitor strain, indicating that both alleles make no noticeable contribution to *V. dahliae* virulence.

## Discussion

Historically, the identification of avirulence genes has been challenging for fungi that reproduce asexually, as genetic mapping cannot be utilized. However, since the advent of affordable genome sequencing, cumbersome and laborious methods to identify avirulence genes, that include functional screenings of fungal cDNAs or protein fractions for the induction of immune responses in plants (Takken *et al*., 2000; Luderer *et al*., 2002), have been supplemented with comparative genomics and transcriptomics strategies (Gibriel *et al*., 2016). Less than a decade ago, we identified the first avirulence gene of *V. dahliae*, known as VdAve1 for mediating **a**virulence on ***Ve1*** plants, through a comparative population genomics strategy combined with transcriptomics by utilizing race 1 strains that were contained by the *Ve1* resistance gene of tomato, and resistance‐breaking race 2 strains (de Jonge *et al*., 2013). In this study, we used a similar approach based on comparative population genomics of race 1 and 2 strains with race 3 strains to successfully identify XLOC_00170 as the Av2 effector that mediates **a**virulence on ***V2*** plants. Intriguingly, besides VdAve1, XLOC_00170 has been identified previously as one of the most highly induced genes of *V. dahliae* during host colonization (de Jonge *et al*., 2013).

*Ve1* and the *V2* locus are the only two major resistance sources that have been described in tomato against *V. dahliae* thus far (Fradin *et al*., 2009; Usami *et al*., 2017). Since its initial introduction from a wild Peruvian tomato accession into cultivars in the 1950s (Deseret News and Telegram, 1955), *Ve1* has been widely exploited as it is incorporated in virtually every tomato cultivar today. Even though soon after the introduction of these cultivars resistance‐breaking race 2 strains emerged, first in the United States (Robinson, 1957; Alexander, 1962), and soon thereafter also in Europe (Cirulli, 1969; Pegg and Dixon, 1969), *Ve1* is still considered useful for Verticillium wilt control today. An important factor that contributes to the durability of resistance is the fitness penalty for the pathogen upon losing the corresponding avirulence factor (Brown, 2015). The VdAve1 effector contributes considerably to *V. dahliae* virulence on tomato, which explains why race 2 strains that lack *VdAve1* are generally less aggressive (de Jonge *et al*., 2012). Based on our current observations that differences in aggressiveness between race 2 and race 3 strains on Moneymaker plants are not obvious (Fig. 1), that genetic complementation of race 3 strains with *Av2* did not lead to a striking increase in aggressiveness on Moneymaker plants (Fig. 3), and that targeted deletion of *Av2* from race 2 strains did not lead to a striking decrease in aggressiveness on Moneymaker plants (Fig. 4), we conclude that the contribution of *Av2* to *V. dahliae* virulence under the conditions tested in this study is modest at most.

Thus far, V2 resistance has been exploited scarcely when compared with Ve1, as it has only been introduced in a number of Japanese rootstock cultivars since 2006 (Usami *et al*., 2017). Previously, *V2* resistance‐breaking race 3 strains have been found in several Japanese prefectures on two separate islands (Usami *et al*., 2017). Intriguingly, our genome analyses demonstrate that race 3 strains that lack *Av2* are ubiquitous and found worldwide, as our collection of sequenced strains comprises specimens that were originally isolated in Europe, China, Canada, and the United States. Arguably, most of these race 3 strains arose in the absence of *V2* selection by tomato cultivation. It is conceivable that, similar to Ve1 homologues that are found in other plant species besides tomato (Song *et al*., 2017), functional homologues of *V2* occur in other plant species as well, which may have selected against the presence of *Av2* in many *V. dahliae* strains. However, as long as *V2* is not cloned this hypothesis cannot be tested.

Like *VdAve1*, *Av2* also resides in an LS region of the *V. dahliae* genome, albeit in another region on another chromosome. Typically, these LS regions are gene‐sparse and enriched in repetitive elements, such as transposons, causing these regions to be highly plastic which is thought to mediate accelerated evolution of effector catalogues (de Jonge *et al*., 2013; Faino *et al*., 2016; Cook, *et al*., 2020). We previously demonstrated that *VdAve1* has been lost from the *V. dahliae* population multiple times, and to date only PAV has been identified as mechanism to escape *Ve1*‐mediated immunity (de Jonge *et al*., 2012, 2013; Faino *et al*., 2016). Similarly, our phylogenetic analysis reveals that *Av2* has been lost multiple times independently, and although we identified two allelic variants, both variants are recognized by V2. Consequently, PAV remains the only mechanism to overcome *V2*‐mediated immunity thus far. Despite the observation that PAV is the only observed mechanism for *V. dahliae* to overcome host immunity, pathogens typically exploit a wide variety of mechanisms, ranging from SNPs (Joosten *et al*., 1994) to altered expression of the avirulence gene (Na and Gijzen, 2016). Nevertheless, avirulence gene deletion to overcome host immunity is common and has been reported for various fungi, including *C. fulvum* (Stergiopoulos *et al*., 2007), *Fusarium oxysporum* (Niu *et al*., 2016; Schmidt *et al*., 2016), *Leptosphaeria maculans* (Gout *et al*., 2007; Petit‐Houdenot *et al*., 2019), *Blumeria graminis* (Praz *et al*., 2016) and *Magnaporthe oryzae* (Pallaghy *et al*., 1994; Zhou *et al*., 2007).

It was previously demonstrated that frequencies of SNPs are significantly reduced in the area surrounding the VdAve1 locus when compared with the surrounding genomic regions (Faino *et al*., 2016), which was thought to point toward recent acquisition through horizontal transfer (de Jonge *et al*., 2012). However, we recently noted that enhanced sequence conservation through reduced nucleotide substitution is a general feature of LS regions in *V. dahliae* (Depotter *et al*., 2019). Although a mechanistic underpinning is still lacking, we hypothesized that differences in chromatin organization may perhaps explain this phenomenon. Interestingly, while DNA methylation is generally low and only present at TEs, only TEs in the core genome are methylated while LS TEs are largely devoid of methylation (Cook *et al*., 2020). Furthermore, TEs within LS regions are more transcriptionally active and display increased DNA accessibility, representing a unique chromatin profile that could contribute to the plasticity of these regions (Faino *et al*., 2016; Cook *et al*., 2020). Possibly, the increased DNA accessibility contributes to the high *in planta* expression of genes residing in these regions, and *VdAve1* as well as *Av2* belong to the most highly expressed genes during host colonization (de Jonge *et al*., 2013).

Our identification of *Av2* concerns the cloning of only the second avirulence gene of *V. dahliae*. This identification may permit its use as a functional tool for genetic mapping of the *V2* gene. Typically, *V. dahliae* symptoms on tomato display considerable variability, and disease phenotyping is laborious. Possibly, injections of heterologously produced Av2 protein can be used to screen tomato plants in genetic mapping analyses, provided that such injections result in a visible phenotype such as a hypersensitive response. Similar effector‐assisted resistance breeding has previously been used successfully identify resistance sources in tomato against the leaf mould pathogen *Cladosporium fulvum* (Lauge *et al*., 1998; Takken *et al*., 1999) and potato against the late blight pathogen *Phytophthora infestans* (Vleeshouwers and Oliver, 2014; Du *et al*., 2015). The identification of *Av2* can furthermore be exploited for race diagnostics of *V. dahliae* to determine whether cultivation of resistant tomato genotypes is useful, but also to monitor *V. dahliae* population dynamics and race structures. Based on the identification of avirulence genes, rapid in‐field diagnostics can be developed to aid growers to cultivate disease‐free crops.

## Materials and methods

### *V. dahliae* inoculation and phenotyping

Plants were grown in potting soil (Potgrond 4, Horticoop, Katwijk, the Netherlands) under controlled greenhouse conditions (Unifarm, Wageningen, the Netherlands) with day/night temperature of 24/18°C for 16‐h/8‐h periods, respectively, and relativity humidity between 50% and 85%. For *V. dahliae* inoculation, 10‐day‐old seedlings were root‐dipped for 10 min as previously described (Fradin *et al*., 2009). Disease symptoms were scored at 21 days post inoculation (dpi) by measuring the canopy area to calculate stunting as follows:

$$\text{stunting}\;\left(\%\right)=\left(1-\frac{\text{canopy area}\;V.\text{dahliae}-\text{inoculated plant}}{\text{average canopy area of mock}-\text{inoculated plants}}\right)*100.$$

To test for significant stunting, an ANOVA was performed which tests for significant differences in canopy area between mock‐inoculated and *V. dahliae* inoculated plants. Outliers were detected based on the studentized residuals from the ANOVA analysis. All datapoints with studentized residuals below −2.5 or above 2.5 were classified as outliers and removed. In total, approximately 1.8% of the datapoints were classified as outlier.

### High‐molecular weight DNA isolation and nanopore sequencing

Conidiospores were harvested from potato dextrose agar (PDA) plates, transferred to Czapek dox medium and grown for 10 days. Subsequently, fungal material was collected on Miracloth, freeze‐dried overnight and ground to powder with mortar and pestle of which 300 mg was incubated for 1 h at 65°C with 350 μl DNA extraction buffer (0.35 M Sorbitol, 0.1 M Tris‐base, 5 mM EDTA pH 7.5), 350 μl nucleic lysis buffer (0.2 M Tris, 0.05 M EDTA, 2 M NaCl, 2% CTAB) and 162.5 μl Sarkosyl (10% w/v) with 1% β‐mercaptoethanol. Next, 400 μl of phenol/chloroform/isoamyl alcohol (25:24:1) was added, shaken and incubated at room temperature (RT) for 5 min before centrifugation at 16 000 g for 15 min. After transfer of the aqueous phase to a new tube, 10 μl of RNAase (10 mg μL^−1^) was added and incubated at 37°C for 1 h. Subsequently, half a volume of chloroform was added, shaken and centrifuged at 16 000 g for 5 min at RT, after which the chloroform extraction was repeated. Next, the aqueous phase was mixed with 10 volumes of 100% ice‐cold ethanol, incubated for 30 min at RT, and the DNA was fished out using a glass hook, transferred to a new tube, and washed twice with 500 μl 70% ethanol. Finally, the DNA was air‐dried, resuspended in nuclease‐free water and incubated at 4°C for 2 days. The DNA quality, size and quantity were assessed by Nanodrop, gel electrophoresis and Qubit analyses.

Library preparation with the Rapid Sequencing Kit (SQK‐RAD004) was performed according to the manufacturer's instructions (Oxford Nanopore Technologies, Oxford, UK) with 400 ng HMW DNA. An R9.4.1 flow cell (Oxford Nanopore Technologies) was loaded and run for 24 h. Base calling was performed using Guppy (version 3.1.5; Oxford Nanopore Technologies) with the high‐accuracy base‐calling algorithm. Adapter sequences were removed using Porechop (version 0.2.4 with default settings; Wick, 2018). Finally, the reads were self‐corrected, trimmed and assembled using Canu (Version 1.8; Koren *et al*., 2017). Sequencing data are available at the NCBI SRA database under accession number PRJNA639910.

### Comparative genomics and candidate identification

Self‐corrected reads from *V. dahliae* race 3 strains were mapped against the reference genome using BWA‐MEM (version 0.7.17; default settings; Li, 2013). Reads with low mapping quality (score < 10) were removed using Samtools view (version 1.9; setting: ‐q 10) (Li *et al*., 2009), and reads mapping in regions with low coverage (<10x) were discarded using Bedtools coverage (version 2.25.0; setting: ‐d) (Quinlan and Hall, 2010). Self‐corrected race 2 strain reads were mapped against the retained reference genome‐specific regions that are absent from the race 3 strains. Retained sequences shared by the reference and every race 2 strain, while absent from every race 3 strain, were retained as *Av2* candidate regions.

The previously determined annotation of *V. dahliae* strain JR2 (Faino *et al*., 2015) was used to extract genes when JR2 or TO22 were used as alignment references. To this end, retained sequences shared by the TO22 reference assembly and race 2 strains, absent from race 3 strains, were mapped against the JR2 genome assembly, and genes in the shared sequences were extracted. The remaining sequences that did not map to the *V. dahliae* strain JR2 genome assembly were annotated using Augustus (version 2.1.5; default settings; Stanke *et al*., 2006). SignalP software (version 4.0; Petersen *et al*., 2011) was used to identify N‐terminal signal peptides in predicted proteins.

### Real‐time PCR

To determine expression profiles of *Av2* candidate genes during *V. dahliae* infection of tomato, 2‐week‐old tomato (cv. Moneymaker) seedlings were inoculated with *V. dahliae* strain JR2 or TO22, and stems were harvested up to 14 dpi. Furthermore, conidiospores were harvested from 5‐day‐old PDA plates. Total RNA extraction and cDNA synthesis were performed as previously described (Santhanam *et al*., 2013). Real time‐PCR was performed with primers listed in Table 3, using the *V. dahliae* glyceraldehyde‐3‐phosphate dehydrogenase gene (*GAPDH*) as endogenous control. The PCR cycling conditions were as follows: an initial 95°C denaturation step for 10 min followed by denaturation for 15 s at 95°C, annealing for 30 s at 60°C, and extension at 72°C for 40 cycles.

**Table 3:** Primers used in this study.

Primer name	Oligonucleotide sequence (5′→3′)	Usage
XLOC00170‐F	CAGCCCTCAATACACCATGAAGATG	qPCR
XLOC00170‐R	TTCCGTGATGCTTCCTACAGAGG	qPCR
evm1569.344‐F	CACTTGCTTGGTTGCATGAT	qPCR
evm1569.344‐R	TCCTTACTGTGCTGGATTCG	qPCR
tig00000058‐cdt1‐F	GAGTGATGCTGTTGGTGTGG	qPCR
tig00000058‐cdt1‐R	AAGTCCGGATTGTCGAACTG	qPCR
tig00000058‐cdt2‐F	CCACACCAACAGCATCACTC	qPCR
tig00000058‐cdt2‐R	CCTGCATCTGCATGTCAAGT	qPCR
tig00000151‐F	TGTCCGCCTCTTCTGACTCT	qPCR
tig00000151‐R	GTTGGCGTGGGTTCTACCTA	qPCR
tig00017428‐F	CCATCCAGACCGAAACAAGT	qPCR
tig00017428‐R	CTGGAAGCCGCAGTTTAGTC	qPCR
VdAve1‐F	AGCTTTCTACGCTTGGA	qPCR
VdAve1‐R	TTGGCTGGGATTGCT	qPCR
VdGAPDH‐F	CGAGTCCACTGGTGTCTTCA	qPCR
VdGAPDH‐R	CCCTCAACGATGGTGAACTT	qPCR
CO‐XLOC00170‐F	cgtctattaattaaATGAAGATGACAATCATAGCTACTTTGGC	Complementation
CO‐XLOC00170‐R	cgtctagcggccgcTTATACGTTAAAGACTACAACGATTAGTAACAATC	Complementation
CO‐Evm344‐F	cgtctattaattaaATGAAACTATCTCTTCCCATTACAGCC	Complementation
CO‐Evm344‐R	cgtctagcggccgcTTAGGAAGCTTTCTTTCGTCCTCG	Complementation
SlRUB‐Fw	GAACAGTTTCTCACTGTTGAC	qPCR
SlRUB‐Rv	CGTGAGAACCATAAGTCACC	qPCR
ITS1‐F	AAAGTTTTAATGGTTCGCTAAGA	qPCR
STVe1‐R	CTTGGTCATTTAGAGGAAGTAA	qPCR
JR2‐XLOC170‐LB‐F	GGTCTTAAUAGAAGAATGTGGTGGGAGGA	Deletion
JR2‐XLOC170‐LB‐R	GGCATTAAUTAGGGAAGGATGGCTGTTTG	Deletion
JR2‐XLOC170‐RB‐F	GGACTTAAUCGAAAGATAGACGTGTTGTTGG	Deletion
JR2‐XLOC170‐RB‐R	GGGTTTAAUACGACGAGAGCCCTTATCAA	Deletion
TO22‐XLOC170‐RB‐F	GGTCTTAAUCAGCGGTAATTTTGCAGTGA	Deletion
TO22‐XLOC170‐RB‐R	GGTCTTAAUGCTGTATTTCCTTGCGCATT	Deletion
TO22‐XLOC170‐LB‐F	GGTCTTAAUTGCGAAGGCAGATGTAACAA	Deletion
TO22‐XLOC170‐LB‐R	GGTCTTAAUTTGAGGGCTGGTTTCGTAGT	Deletion

### Genome mining

In total, 44 previously sequenced *V. dahliae* strains and eight strains sequenced in this study were mined for *Av2* gene candidates using BLASTn. Gene sequences were extracted using Bedtools (setting: getfasta) (Quinlan and Hall, 2010) and aligned to determine allelic variation using Espript (version 3.0; default settings) (Robert and Gouet, 2014). Similarly, amino acid sequences were aligned using Espript (Robert and Gouet, 2014).

To determine the genomic localization of *XLOC_00170* and *Evm_344*, the *V. dahliae* strain JR2 assembly and annotation were used (Faino *et al*., 2015) together with coverage plots from reads of race 3 and race 2 strains as described in comparative genomics approach IV (Table 2) using R scripts, with the package karyoploteR for R (version 3.6) using kpPlotBAMCoverage function. The schematic representation of the genomic region on chromosome 4 with *XLOC_00170* and *Evm_344* was generated using Integrative Genomics Viewer (IGV) software v2.6.3 (Robinson *et al*., 2011) and R package (version 3.6) Gviz (Hahne and Ivanek, 2016).

### Phylogenetic tree construction

The phylogenetic tree of 52 *V. dahliae* strains was generated with Realphy (version 1.12) (Bertels *et al*., 2014) using Bowtie2 (version 2.2.6) (Langmead and Salzberg, 2012) to map genomic reads against the *V. dahliae* strain JR2 assembly. A maximum likelihood phylogenetic tree was inferred using RAxML (version 8.2.8) (Stamatakis, 2014).

### Presence–absence variation analysis

Presence–absence variation (PAV) was identified by using whole‐genome alignments for 17 *V. dahliae* strains. Paired‐end short reads were mapped to *V. dahliae* strain JR2 (Faino *et al*., 2015) using BWA‐mem with default settings (Li and Durbin, 2009). Long‐reads were mapped using minimap2 with default settings (Li, 2018). Using the Picard toolkit (http://broadinstitute.github.io/picard/), library artefacts were marked and removed with ‐*MarkDuplicates* followed by *‐SortSam* to sort the reads. Raw read coverage was averaged per 100 bp non‐overlapping windows using the BEDtools *‐multicov* function (Quinlan and Hall, 2010). Next, we transformed the raw read coverage values to a binary matrix by applying a cut‐off of 10 reads for short‐read data; > = 10 reads indicate presence (1) and < 10 reads indicate absence (0) of the respective genomic region. For long‐read data a cut‐off of 1 read was used; > = 1 read indicates presence (1) and < 1 read indicates absence (0). The total number of PAV counts for each of the 100 bp genomic windows within 100 kb upstream and downstream of the candidate effectors was summarized.

### Genetic complementation, deletion and functional analysis

For genomic complementation of race 3 strains GF‐CB5 and HOMCF, a genomic construct was generated comprising the coding sequence of *XLOC_00170* or *Evm_344* in vector pFBT005 behind the *VdAve1* promoter, using primers CO‐XLOC00170‐F and CO‐XLOC00170‐R for *XLOC_00170* or CO‐Evm344‐F and CO‐Evm344‐R for *Evm_344* (Table 3).

For genomic deletion of *XLOC_00170* from JR2Δ*Ave1* and race 2 strain TO22, a genomic construct was generated comprising the flanking regions of *XLOC_00170* in vector pRF‐NU2 (for JR2Δ*Ave1*) or pRF‐HU2 (for strain TO22), using primers JR2‐XLOC170‐LB‐F, JR2‐XLOC170‐LB‐R, JR2‐XLOC170‐RB‐F and JR2‐XLOC170‐RB‐R for strain JR2, and primers TO22‐XLOC170‐LB‐F, TO22‐XLOC170‐LB‐R, TO22‐XLOC170‐RB‐F and TO22‐XLOC170‐RB‐R for strain TO22 (Table 3).

*Agrobacterium tumefaciens*‐mediated transformation (ATMT) was performed as described previously (Ökmen *et al*., 2013) with a few modifications. *A. tumefaciens* was grown in 5 ml minimal medium (MM) supplemented with 50 μg m^−1^ kanamycin at 28°C for 2 days. After subsequent centrifugation at 3000 g (5 min), cells were resuspended in 5 ml induction medium (IM) supplemented with 50 μg m^−1^ kanamycin, adjusted to OD_600_ 0.15 and grown at 28°C for minimum 6 h until OD_600_ 0.5. Simultaneously, conidiospores of *V. dahliae* race 3 strains GF‐CB5 and HOMCF were harvested after 1 week of cultivation on PDA plates with water, rinsed, and adjusted to a final concentration of 10^6^ conidiospores mL^−1^. The *A. tumefaciens* suspension was mixed with *V. dahliae* conidiospores in a 1:1 volume ratio and 200 μl of the mixture was spread onto PVDF membranes in the centre of IM agar plates. After 2 days at 22°C, membranes were transferred to fresh PDA plates supplemented with 20 μg m^−1^ nourseothricin and 200 μM cefotaxime and incubated at 22°C for two weeks until *V. dahliae* colonies emerged. Transformants that appeared were transferred to fresh PDA supplemented with 20 μg ml^−1^ nourseothricin and 200 μM cefotaxime. Successful transformation was verified by PCR and DNA sequencing.

*V. dahliae* inoculations were performed as described previously (Fradin *et al*., 2009). Disease symptoms were scored 14 days after inoculation by measuring the canopy area to calculate stunting when compared with mock‐inoculated plants. Outgrowth of *V. dahliae* from stem slices was assessed as described previously (de Jonge *et al*., 2012). For biomass quantification, stems were freeze‐dried and ground to powder, of which ∼100 mg was used for DNA isolation. Real‐time PCR was conducted with primers SlRUB‐Fw and SlRUB‐Rv for tomato *RuBisCo* and primers ITS1‐F and STVe1‐R for *V. dahliae* ITS (Table 3). Real‐time PCR conditions were as follows: an initial 95°C denaturation step for 10 min followed by denaturation for 15 s at 95°C and annealing for 30 s at 60°C, and extension at 72°C for 40 cycles.

## Author contributions

J.P.V., T.U., M.F.S. and B.P.H.J.T. conceived the study; E.A.C.C., J.P.V., D.T., M.F.S. and B.P.H.J.T. designed experiments; E.A.C.C., J.P.V., D.T. performed experiments; E.A.C.C., J.P.V., D.T., H.J.S., Y.B., M.F.S. and B.P.H.J.T. analysed data, E.A.C.C., J.P.V. and B.P.H.J.T. wrote the manuscript; MFS and BPHJT supervised the project, all authors discussed the results and contributed to the final manuscript.
